# Can This Dog Be Rehomed to You? A Qualitative Analysis and Assessment of the Scientific Quality of the Potential Adopter Screening Policies and Procedures of Rehoming Organisations

**DOI:** 10.3389/fvets.2020.617525

**Published:** 2020-12-23

**Authors:** Karen E. Griffin, Elizabeth John, Tom Pike, Daniel S. Mills

**Affiliations:** ^1^School of Life Sciences, College of Science, University of Lincoln, Lincoln, United Kingdom; ^2^College of Science, University of Lincoln, Lincoln, United Kingdom

**Keywords:** adopter screening, dog adopter, rehoming, rehoming organisation, shelter

## Abstract

Unwanted dogs are an international problem, and rehoming organisations are tasked with finding many of them appropriate homes. Whilst the processes involved in assessing dogs' suitability for rehoming have received considerable academic attention, the policies and procedures organisations employ for screening potential adopters, which are equally as important to dogs' outcomes, appear to be largely overlooked. Therefore, the aim of this study was to conduct a qualitative analysis of rehoming organisations' adopter screening processes in order to gain insight into what is being done, the extent to which this appears to have any scientific rationale, and what other factors might be driving the process. A written enquiry was sent to organisations in the UK; topics addressed included whether they use a standardised screening process, whether they interview potential adopters and what information is gathered during the interview, and how they score responses. Information was received from 82 respondents. Pre-adoption home visits were the most commonly used method. Self-administered questionnaires were the most standardised method. Using a thematic analysis, ten themes emerged from the types of information gathered during the screening process; 31 characteristics could lead an adopter being deemed unsuitable to adopt a dog. Evidence to potentially support these was found for only eight of them in the academic literature relating to risk factors for relinquishment and human safety risk. The inclusion of some of the characteristics considered important was thought to be for the purpose of ensuring a good quality of life for a dog, but there is a lack of relevant research investigating this. Organisations seem to invest considerable resources into screening potential adopters, but there is limited scientific, and sometimes logical, rationale for this. A further concern relates to the quality of the assessment processes, which show little evidence of quality control measures. Until the necessary research is conducted, it could be argued, from a pragmatic perspective, that organisations should relax their strict screening criteria, and focus their resources on ensuring owners are fully prepared for the changes in their life associated with the inclusion of a new dog in their home and supporting them as necessary.

## Introduction

Many rehoming organisations engage in some form of screening of potential adopters with a view to increasing the likelihood of a successful adoption; however there remain significant gaps in our knowledge of the process. The development of dog assessment procedures prior to rehoming have formed the focus of much research in this area [e.g., (1, 2)], with a growing interest in the need to consider the quality (reliability and validity) of these procedures, as outlined by Taylor and Mills (3) [e.g., (4–6)]. However, it is argued that the quality of such tests remains poor (7) and that they are unlikely to ever be adequate (8). Another approach is to identify and examine risk factors for relinquishment relating to both dogs and adopters, and to create policies based around these which minimise the risk of a dog ending up in a “high risk” situation. The research underpinning this information is often done either retrospectively by contacting surrendering owners after they have relinquished a dog, or by collecting data from surrendering owners at the point of relinquishment [e.g., (9–11)]. However, the quality of such information is subject to bias from both the lapsing of time and social desirability bias, especially as the act of relinquishing a dog can be both emotionally charged and have a negative stigma attached to it (12). Furthermore, as noted by Patronek et al. (9), retrospective study designs in this field may struggle to establish causal relationships. Prospective studies that overcome these issues are rare, although Diesel et al. (13) using a prospective cohort study design, have tracked the outcomes of a sample of dogs adopted from multiple rehoming centres over a 1-year period. While they did identify some new risk factors, such as the presence of children <13 years old in the home, many of their findings reaffirmed the importance of those previously reported in retrospective studies; such as behavioural problems being the most common reason for return [e.g., (14, 15)]. Diesel et al. (13) also reported a return rate of rehomed dogs of 14.7%, which is close to the 15.1% reported by Marston et al. (16) for three Australian shelters. This suggests that there is still some considerable problem with finding the right home for a given dog in the long term.

In addition to the research that has investigated risk factors for relinquishment, there are two other areas of scientific research that need to be considered to further minimize the likelihood that a dog will end up in a “high risk” situation: the work on the risk of human injury from dogs, and that looking into the factors affecting a dog's quality of life or overall wellbeing. Indeed it is not known how or to what extent the scientific information available is being used in practice. Ultimately these policy decisions can have as much impact as a failed test, but this important part of the process seems to have received much less research attention, and we know little about what policies are in place and why. Therefore, the aim of this study was to conduct a qualitative analysis of rehoming organisations' adopter screening policies and procedures in order to gain insight into what is being done by shelters, the extent to which this appears to have any scientific rationale, and what other factors might be driving the process. The results provide important insight into current practice across the shelter/rescue sector, the extent to which current scientific understanding is being integrated into rehoming practice as well as insight into future scientific challenges for those researching this area.

## Materials and Methods

A list of dog rehoming organisations in the UK was compiled from the Association of Dogs and Cats Homes (ADCH) website (www.adch.org.uk). All organisations listed as full or associate members of the ADCH as of July 2012 were contacted electronically and/or via post. A total of 269 organisations and respective branches or centres from across the UK were contacted. This was comprised of 93 separate organisations, six of which had between two and 96 branches. In the written enquiry, organisations were asked about their policies and procedures employed to screen potential adopters; namely:

1. “Do you have standardized questionnaires or criteria employed across the organisation for the adoption process, or do they vary from location to location? If you have a generic document, would you be willing to please send me a copy of it? Alternatively, if you have local procedures, would you please put me in touch with the relevant local contacts?
2. Do you conduct an interview with potential adopters or do they only complete a form that gathers their information? If you conduct an interview, what questions do you ask, and are they consistent from adoption to adoption?
3. How do you judge or score the responses given either via a questionnaire or interview? For example, are the responses to some questions given more value than others, such as the amount of time that an adopter is away from home during the day, or if they live in an apartment vs. a house with a garden? Please provide as much detail as you can.
4. Do you require that you meet all members of the adopter's family who will be living with the dog, or at least have some form of contact with them? If so, for what purpose?
5. Do you conduct a home visit prior to adoption? If so, are there specific criteria that must be met in order for an adoption to be approved? What are the details of this please?”

Organisations were additionally requested to provide supplementary material electronically or via post if possible (e.g., questionnaires, forms, etc.). Organisations were contacted between 30 August 2012 and 18 March 2013.

The data collected from the organisations (*data corpus)* (17) was divided into three categories, collated on an Excel spreadsheet:

1. self-administered questionnaires (separate from home visit forms completed by staff/volunteers)
2. interviews (separate from interviews conducted at a home visit)
3. pre-adoption home visits.

Post-adoption visits were not evaluated as the current study was focused on what happens up to the point of adoption. For each of the categories, columns were created to note three additional criteria:

- whether each respondent employed the particular screening procedure,
- whether the procedure was standardised from case to case, and
- whether the items or topics addressed were known.

In addition for the interviews and home visits it was noted who (e.g., staff or volunteer) was responsible for conducting them. Organisations were also asked how responses from the screening procedures are scored or judged. Of particular note was whether an organisation has specific, fixed criteria that must be met for an adopter to be deemed eligible to adopt any dog (*necessary criteria*) and whether any criteria or collection of criteria were adequate alone for acceptance (*sufficient criteria*). The former type of protocol was labelled *pass/fail scoring*. The specific, necessary criteria that each organisation uses for their pass/fail scoring was recorded and differentiated from “high value criteria” (i.e., what is preferred, but not mandatory). Necessary criteria were identified either by organisations explicitly stating that it was required, or by the usage of the word *must* in their responses (e.g., must have a garden).

A thematic analysis was then undertaken using the procedural framework outlined by Braun and Clarke (17) to create the *data set*; this included only the information deemed relevant for analysis to achieve the current study's aim. Themes were as defined by Braun and Clarke (17), i.e., “A theme captures something important about the data in relation to the research question, and represents some level of *patterned* response or meaning within the data set.”

The analysis was conducted in relation to addressing four key research questions (RQs), which might give insight into the culture underpinning assessment policy:

RQ1. What information or characteristics about an adopter are reported as “most important”? (“Most important” characteristics were those that organisations rated as high value criteria, i.e., that which is preferred, but not mandatory to adopt a dog.)
RQ2. What information or characteristics about an adopter would lead them to be deemed unable to adopt a dog?
RQ3. What evidence is in the scientific literature to support the inclusion of the “most important” characteristics as part of adopter screening assessments?
RQ4. How are adopter screening assessments implemented at a practical level?

In order to address the first two questions, a “bottom up” or inductive approach was applied to the analysis of responses. RQ1 was addressed simply through the collation of data as described below and the second through the focused identification of organisations' implementation of a pass/fail scoring system, and the necessary criteria for an adopter to be deemed eligible to adopt a dog. The “bottom up” approach consisted of identifying items and topics pertaining to similar attributes or factors. These were then grouped to form a theme. Each theme was then named and defined based on the attributes or factors that it encompassed, since it was not necessarily obvious how one theme varied from another based solely on their names. Once themes were determined, sub-themes were generated from an assessment of what the organisations reported giving more weight to during assessments, i.e., the factors determined to be “most important.” Using this portion of the data set, sub-themes were determined on the basis of two criteria:

1. the frequency of responses referring to a sub-theme (e.g., maximum amount of time that a dog is permitted to be left home alone during the day), or
2. a required factor that would determine whether or not the adopter is deemed eligible to adopt a dog (e.g., no laminate flooring in main living areas of residence). In this case, these factors may only have been stated by one organisation in the sample, but their necessity in the screening process warranted them becoming a sub-theme in their own right.

Creating sub-themes was a multi-stage process, which involved some redundancy in reading and re-reading this portion of the data set. This was done to identify the above criteria to establish tiers of sub-themes. Three tiers of sub-themes were established; the tiers progressed from broader concepts (e.g., a garden), to specific characteristics about that concept (e.g., a garden with a secure five-foot fence). Subsequent tiers were created based on the specificity of factors determined by the two criteria outlined above; a sub-theme based on either of these criteria could have resided in any of the tiers (e.g., a required factor to be eligible to adopt a dog may have been the first or the third tier). This process generated the data required to address RQ1 and RQ2. However, not all themes contained sub-themes, e.g., when there were no necessary criteria to adopt a dog as part of the theme. Similarly, those themes that did contain sub-themes did not all necessarily contain three tiers. The level of specificity was the criterion separating tiers.

In order to address RQ3, to determine if there is any scientific basis (i.e., evidence) for the inclusion of the factors that are addressed or the types information sought during adopter screening assessments, the scientific literature was reviewed for three purposes:

1. to identify whether any statistically significant increased risks for relinquishment were associated with these factors,
2. to identify whether any of these factors were statistically associated with a dog's quality of life or overall welfare, and
3. to identify whether any of these factors could be associated with an increased risk to human safety.

A literature search was conducted online using various databases and search engines, including ScienceDirect, Wiley Online Library, and Google Scholar. Keywords used in this search included: *dog adoption, dog relinquishment, dog rehoming, shelter dogs*, and *animal shelters*. Articles that were not available online, such as for older publications, were accessed in journals' printed versions through the University of Lincoln library, or were requested via interlibrary transfer from The British Library. The scientific literature reviewed for the first purpose focused on characteristics of surrendering owners and their dogs, and reasons reported by owners for surrendering their dogs; any published studies with this focus were included, regardless of factors such as sample size and location of the study. Those that mentioned factors, but did not report an increased risk for relinquishment associated with them, such as descriptive reports and opinion pieces, were noted but were not considered scientific evidence. Included within this body of literature are studies of rehoming success (i.e., dogs that have remained in a home) and dog relinquishment (see Table 1) (There is variation in how the terms *dog relinquishment* and *dog return* are used in the relevant studies; whether or not the dog is adopted from and surrendered to the same shelter or rehoming organisation is often the differentiating characteristic, but other variables, such as the amount of time that has lapsed between adoption and surrender, may determine the terminology used [e.g., (10)]. Those studies involving samples of dogs that were adopted from and surrendered to the same organisation are noted accordingly in Table 1) The scientific literature reviewed for the second purpose investigated whether a series of owner and dog characteristics were associated with a good quality of life for a dog in a non-clinical population (e.g., dogs that were not ill); any published studies with this focus were included (see Table 1). For the third purpose, all factors included in assessments were reviewed to determine which factors may be included for the purpose of mitigating a risk to human safety. The scientific literature used for this purpose focused on reviewing incidence rates of humans who had suffered dog bites. Depending on the study, data was collected in various manners (e.g., reviewing hospital admission records, telephone interviews), and either included bites on any region of the body or to a specific area (e.g., the head). Similar to the literature reviewed for the first purpose, those studies that did not report any statistical significance were noted but were not included as scientific evidence (see Table 1).

**Table 1:** Scientific literature reviewed to identify whether factors included in adopter screening assessments are statistically associated with an increased risk for relinquishment, with a dog's quality of life, or a risk to human safety.

**Study**	**Sample size(s)**	**Location of study**	**Included for which purpose**	**Source(s) of data**
Carter and Taylor (18)	*n* = 117^a^	Australia	Risk for relinquishment	• Retrospective analysis of shelter intake forms
				• Additional questionnaire administered (as part of the study) to surrendering owners at the point of relinquishment
				• Semi-structured interviews at the point of relinquishment
Chen et al. (19)	*n* = 537	US	Risk to human safety	Review of paediatric patients' hospital medical records who had suffered a facial dog bite
Diesel et al. (13)	*n* = 662	UK	Risk for relinquishment^b^	• Veterinary records and behavioural assessments (from the rehoming organisation involved in the study)
				• Questionnaire completed by dog relinquishers via post 6–8 weeks post-relinquishment^c^
				• Telephone call 6 months post-adoption to ensure new owner still had the dog
Diesel et al. (4)	*n* = 2,806	UK	Risk for relinquishment	Questionnaire completed (as part of the study) by relinquishing owners at the point of relinquishment
Dolan et al. (20)	*n* = 166	US	Risk for relinquishment	Survey administered (as part of the study) to surrendering owners at the point of relinquishment
Fuh et al. (21)	*n* = 229	Taiwan	Risk for relinquishment	Telephone survey with surrendering owners post-relinquishment
Gilchrist et al. (22)	*n* = 5,638	US	Risk to human safety	Randomised telephone survey (government sponsored)
Horswell and Chahine (23)	*n* = 40	US	Risk to human safety	Review of paediatric patients' hospital medical records who had suffered a dog bite to the face, neck, or head
Kwan and Bain (24)	*n* = ~80	US	Risk for relinquishment	Survey administered (as part of the study) to surrendering owners at the point of relinquishment
Marinelli et al. (25)	*n* = 104	Italy	Quality of life	• Three questionnaires administered to dog owners
				• Physical examination of the dogs
				• Strange Situation Test
				• Lexington Attachment to Pets Scale
Marston et al. (16)	*n* = 3,123^d^	Australia	Risk for relinquishment	Shelter records for admitted dogs
Mondelli et al. (15)	*n* = 307	Italy	Risk for relinquishment^a^	Survey administered (as part of the study) to surrendering owners at the point of relinquishment
New et al. (26)	*n* = 2,631	US	Risk for relinquishment	Structured interview with dog relinquishers post-relinquishment^c^
Patronek et al. (9)	*n* = 285	US	Risk for relinquishment	Structured telephone interview with dog relinquishers post-relinquishment^e^
Patronek et al. (27)	*n* = 256	US	Risk to human safety	Interviews with employees of law enforcement agencies
Salman et al. (28)	*n* = 3,676	US	Risk for relinquishment	Questionnaire administered (as part of the study) to surrendering owners at the point of relinquishment
Scarlett et al. (11)	*n* = 2,045	US	Risk for relinquishment	Questionnaire administered (as part of the study) to surrendering owners at the point of relinquishment
Scarlett et al.(29)	*n* = 341	Austria	Risk to human safety	Review of paediatric patients' hospital medical records
Shore (10)	*n* = ~100	US	Risk for relinquishment^b^	• Form completed by surrendering owners (routine form used by shelter)
				• Adoption records from shelter
				• Telephone interview with dog relinquishers post-relinquishment^d^
Shuler et al. (30)	*n* = 636	US	Risk to human safety	Review of dog bite injury records from municipal animal control office
Vućinić et al. (31)	*n* = 156^f^ *n* = 1,005^g^	Serbia	Risk for relinquishment	Questionnaire administered (as part of the study) to surrendering owners at the point of relinquishment
Weiss et al. (32)	*n* = 333,687	US	Risk to human safety	Government survey of hospital emergency department patient cases
Weiss et al. (33)	*n* = ~150	US	Risk for relinquishment	Survey administered (as part of the study) to surrendering owners at the point of relinquishment

a *The study reported the total sample size for owners relinquishing companion animals (dogs and cats), but it did not report the sample size for just those relinquishing dogs*.

b *The sample was comprised of dogs adopted from and returned to the same shelter or rehoming organisation*.

c *The study was completed prospectively; the sample was comprised of dogs relinquished to the rehoming centre who were then rehomed*.

d *The study included all dogs admitted to the shelter; this sample size is just for owner relinquished dogs*.

e *The amount of time that lapsed between the point of the relinquishment and the interview was not reported*.

f *Dogs relinquished for adoption/rehoming*.

g *Dogs relinquished for euthanasia*.

In order to address RQ4, the responses pertaining to how adopter screening assessments are practically executed were evaluated. This included how respondents score or judge potential adopters' responses, who is responsible for conducting home visits and interviews (for those respondents that use them), and the level of standardisation of screening methods.

## Results

Responses that included information about adopter screening policies and procedures were received from 82 respondents, 30.5% of the sample of organisations to which the written enquiry was sent. Pre-adoption home visits were the most commonly used adopter screening method, followed by interviews, and self-administered questionnaires (Table 2). Not all respondents provided information about what adopter factors or characteristics are addressed in each screening method. Of the 81 that use them, 54 respondents (66.67%) provided information about what factors are addressed in pre-adoption home visits. Of the 67 that use them, 53 respondents (79.10%) provided information about factors addressed in self-administered questionnaires. Of the 69 that use them, 30 respondents (43.48%) provided information about factors addressed in interviews.

**Table 2:** Frequency of adopter screening methods used by respondents (*n* = 82).

	**Always used**	**Sometimes used**	**Never used**	**(No info)**
Pre-adoption home visits	73 (89.02%)	8 (9.76%)	1 (1.22%)	0
Interviews	68 (82.93%)	1 (1.22%)	3 (3.66%)	10 (12.20%)
Self-administered questionnaires	67 (81.71%)	0	11 (13.41%)	4 (4.88%)

### RQ1: What Information or Characteristics About an Adopter Are Reported as “Most Important”?

**Figure 1:**
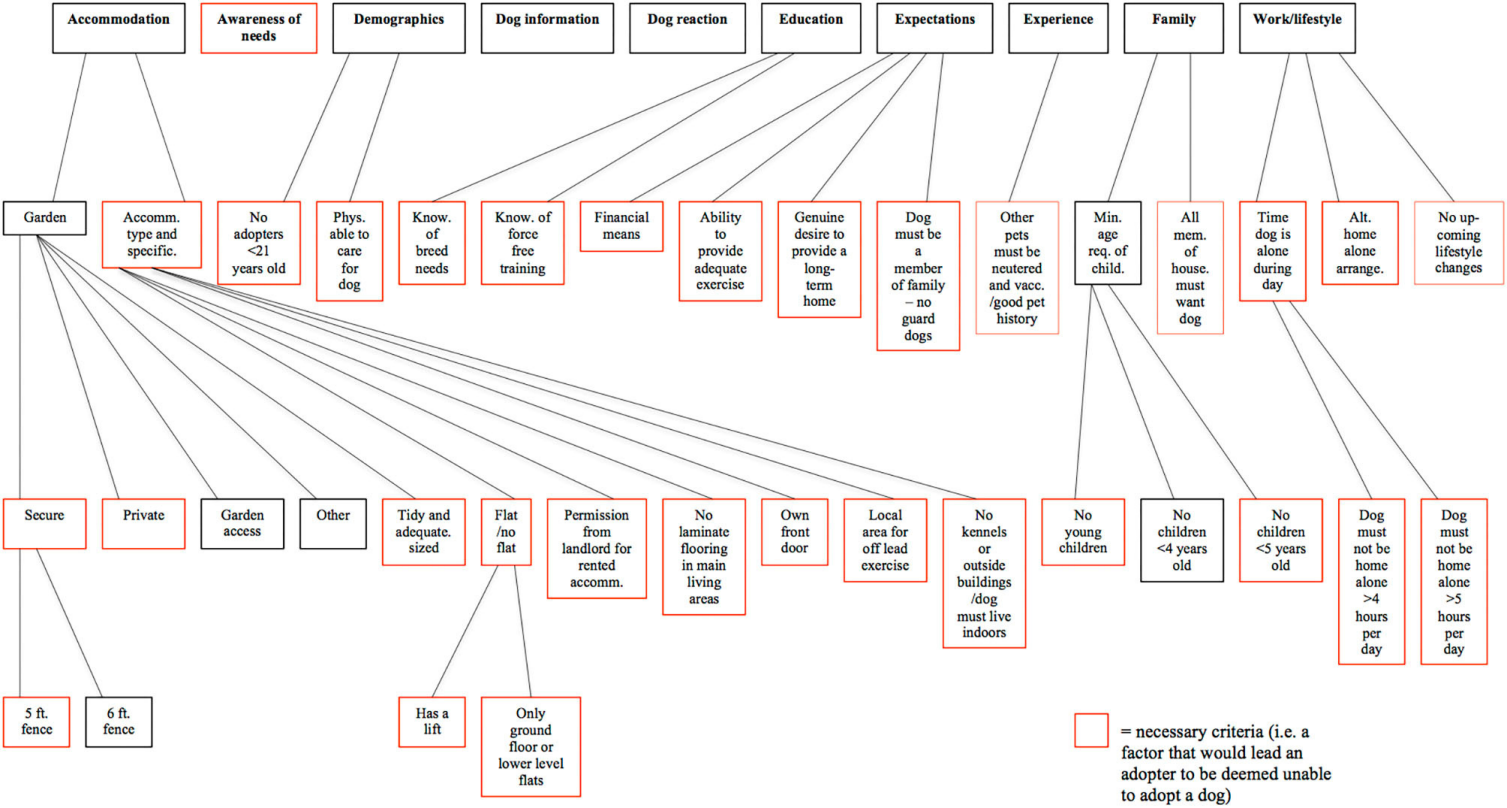
Adopter screening item themes and sub-themes.

Ten themes emerged: *accommodation, awareness of needs, demographics, dog information, dog reaction, education, expectations, experience, family*, and *work/lifestyle*. The definition for each is given in Table 3. Seven of the 10 themes contained sub-themes (see Figure 1). Each sub-theme presented at least one “most important” characteristic of a potential adopter as defined in this study (see Materials and Methods section). One theme, *awareness of needs*, was, in itself, a “most important” characteristic. Sub-themes were comprised of both objective, measurable factors (e.g., garden fence height), and subjective factors (e.g., adopter must have a genuine desire to provide a long-term home for a dog). A total of 36 sub-themes were created spanning three tiers, though not all themes contained that many tiers. *Accommodation* had both the greatest number of sub-themes and the most tiers, followed by *family*. *Awareness of needs, dog information*, and *dog reaction* did not have any sub-themes (Table 4). Aspects of the latter two sub-themes were included in the adopter screening process, which is why the themes exist, but characteristics pertaining to them were not reported by organisations as either “most important” or something that would lead an adopter being deemed unable to adopt a dog.

**Table 3:** Themes present, their definition, and prevalence in self-administered questionnaires, interviews, and pre-adoption home visits.

**Theme**	**Definition**	**Themes present in each screening method**		
		**Questionnaires**	**Interviews**	**Pre-adoption**
				**home visits**
Accommodation	• The type of accommodation in which the adopter lives (e.g., house, flat), and the nature of the housing (e.g., council, HM Forces), and if there is garden access, and if so is it enclosed?	√	√	√
	• If the accommodation is rented, the organisation may require written approval from the landlord that a dog is permitted (e.g., tenancy agreement or letter)			
Awareness of needs	The adopter's awareness of dogs' needs, and their preparedness to meet such needs, often specifically focusing on the needs of the particular dog (e.g., the cost of veterinary care for the dog's chronic health condition)			√
Demographics	The adopter's name, address, contact info, and age	√	√	
Dog information	• What sort of dog the adopter is seeking (e.g., sex, breed, size, and age)	√	√	√
	• Specific desired characteristics of a dog (e.g., friendly with other dogs, good when left alone)			
	• The identifying information of a particular dog the adopter has in mind that is part of the organisation (e.g., a dog they have seen on the organisation's website)			
Dog reaction	Gauging the potential adoptee dog's reaction to family members, their accommodation, and overall new environment by bringing the dog along on a home visit			√
Education	Educating the adopter and other members of the household about responsible dog ownership (e.g., proper handling, training, and general care of a dog)			√
Expectations	The adopter's expectations of having a dog in general, including vet and other related costs, responsibilities of having a dog (e.g., amount of daily exercise to be provided)	√	√	
Experience	The adopter's current and past experience with dogs (e.g., do they currently have a dog, and if so is the dog neutered and vaccinated, vet reference)	√	√	√
Family	• The adopter's family structure (e.g., children in the household, other animals in the household besides dogs), and history of family members' medical issues associated with dogs (e.g., allergies) • In the case of home visits, some organisations may require all family members living in the household to be present	√	√	√
Work/lifestyle	The nature of the adopter's job (e.g., full time, hours worked per day, time dog would be left alone daily, etc.), and other upcoming events (e.g., planned holiday, expecting a baby, moving house)	√	√	√

**Table 4:** Number of “most important” characteristics of a potential adopter by theme.

**Theme**	**Number of characteristics**	**Number of**
	**(sub-themes)**	**tiers**
Accommodation	17	3
Family	5	2
Work/lifestyle	5	2
Expectations	4	1
Demographics	2	1
Education	2	1
Experience	1	1
Awareness of needs^a^	0	
Dog information	0	
Dog reaction	0	

a *Awareness of needs as a theme was a “most important” characteristic itself*.

### RQ2: What Information or Characteristics About an Adopter Would Lead Them to be Deemed Unable to Adopt a Dog?

Within the themes are sub-themes that represent characteristics of a potential adopter that would lead them to be deemed unable to adopt a dog by some organisations. Not all organisations screen potential adopters in this manner and have such criteria. Forty respondents (48.78%) were identified as having this scoring system; 35 respondents (42.68%) did not have it and seven respondents (8.54%) did not provide any information about how they judge or score adopters' responses during the screening process.

Thirty-one characteristics about an adopter were identified by at least one respondent as preventing adoption of a dog (see Figure 1). The only themes that contained characteristics that were highly valued but not required were *accommodation* and *family*. The majority of the “most important” characteristics were also deemed to be features that could prevent adoption of a dog, but possibly only by one organisation (e.g., “knowledge of breed needs”). Some characteristics were preferable but not required for other respondents (e.g., “no young children”), but because they were reported as a requirement by at least one respondent they are included in this subset (see Table 5).

**Table 5:** Respondent frequency of “preferred” vs. “required” “most important” characteristics.

**Characteristic**	**Number of respondents as preferred characteristic**	**Number of respondents as required characteristic**
Ability to provide adequate exercise	1	1^a^
Accommodation type and specification	1	1
Flat/no flat	4	2
Has a lift	0	2
Only ground floor or lower level flats	0	5^b^
Local area for off-lead exercise	0	1
No laminate flooring in main living areas of house	0	1
No kennels or outside buildings/dog must live indoors	1	8
Own front door	0	1
Permission from landlord for rented accommodation	0	4
All members of household must want dog	0	1
Alternate home alone arrangements for dog	3	7^a^
Amount of time dog is alone during day	13	16
Dog must not be home alone for >4 h per day	1	6
Dog must not be home alone for >5 h per day	1	4
Awareness of needs	0	1
Dog must be member of family—no guard dogs	0	4
Financial means to care for dog	0	2
Garden	12	17
Garden access	5	0
Other (features of garden)	2	0
Private garden	0	4
Secure garden	4	13
5 foot fence	0	2
6 foot fence	2	0
Tidy and adequately sized garden	0	1
Genuine desire to provide a long-term home for a dog	0	1
Knowledge of breed needs	3	1
Knowledge of force free training	0	1
Minimum age requirement of children in household	2	0
No children <4 years old	1	0
No children <5 years old	2^c^	4^c^
No young children	7	3^d^^,^^e^
No adopters <21 years old	0	1
No upcoming lifestyle changes	0	1
Other pets must be neutered and vaccinated/good pet history	3	4
Physically able to care for dog	0	1

a *For one respondent only if the adopter lives in a flat*.

b *One respondent will only rehome small dogs to adopters living in flats*.

c *For one respondent only applies to puppies being rehomed*.

d *For one respondent only applies if young children will be left alone with dog for long periods of time*.

e *For one respondent only applies if a dog's history is unknown*.

### RQ3: What Evidence Is in the Scientific Literature to Support the Inclusion of the “Most Important” Characteristics as Part of Adopter Screening Assessments?

Evidence was found in the scientific literature to support the inclusion of eight of the 37 “most important” characteristics in adopter screening assessments on the basis of three reasons with statistically significant associations: an increased risk for relinquishment, a dog's quality of life or overall welfare, and a risk to human safety (see Table 6). There was evidence in both the literature pertaining to relinquishment risk and human safety risk for four of the characteristics, which were all part of the *family* theme. Four studies reported a significant increased risk of human injury associated with child age (19, 29, 30, 32), and one study (13) reported a significantly increased risk for relinquishment associated with the age of children. An additional three studies mentioned these characteristics (22, 23, 27), but they did not provide statistical evidence to support their claim.

**Table 6:** “Most important” characteristics that are mentioned in the literature as reasons for relinquishment/characteristics of surrendering owners, in relation to human safety risk, or in relation to a dog's quality of life/overall welfare.

**Theme**	**Reason/characteristic**	**Total number of studies mentioned in**	**Number of studies with reported evidence^a^**	**Associated with an increased risk for relinquishment**	**Associated with a risk to human safety**	**Associated with a dog's quality of life/overall welfare**
Family	Minimum age requirement of children in the household	9	5	√	√	
Family	No children <4 years old	9	5	√	√	
Family	No children <5 years old	9	5	√	√	
Family	No young children	9	5	√	√	
Expectations	Financial means	6	2	√		
Accommodation	Living in a flat or apartment	5	2	√		
Demographics	No adopters <21 years old	5	2	√		
Accommodation	No kennels or outside buildings/dog must live indoors	1	1	√		
Work/lifestyle	Lifestyle changes	9				
Awareness of needs	Awareness of needs	6				
Family	All members of household must want dog	3				
Work/lifestyle	Amount of time dog is alone during day	2				
Accommodation	The presence of a garden	2				
Accommodation	Landlord issues	2				
Accommodation	Secure garden	1				
Experience	Experience^b^	1				

a *Statistically significant evidence for any of the three reasons (i.e., an increased risk for relinquishment, a risk to human safety, a dog's quality of life)*.

b *A theme (though not a “most important” characteristic) mentioned in the literature in relation to a dog's quality of life*.

Schalamon et al. (29) reported that the highest incidence of dog bites was in 1-year old children, with the incidence decreasing thereafter with age; 73% of children (248/341) bitten were younger than 10 years old. The study reported that children who sustained dog bites to their head and neck were significantly younger compared with the total study population (i.e., 0–16 years) with a mean age of 4.1 years old (*p* < 0.01). The study also reported that children who were younger than 5 years sustained significantly more dog bite attacks by small dogs compared with older children (*p* = 0.04). Similarly, Shuler et al. (30) reported that the rate of dog bites sustained by boys aged 5–9 years old was significantly higher than the rate of other male age categories (*p* = 0.01), and had the highest incidence rate of any other sex/age category (178 per 100,000 children). In an analysis of children who sought medical attention for a facial dog bite, Chen et al. (19) reported that the majority occurred in children 0–5 years old (68% [365/537]), and the highest incidence occurred in 3-year old children (15.8% [85/537]). The authors noted that the incidence rate decreased with increasing age. The study also reported that children 0–5 years old and 6–12 years old were significantly more likely to have known the dog that bit them (*p* < 0.0001; *p* = 0.0018, respectively). Diesel et al. (13) reported that dogs rehomed to families with children ≤13 years old were statistically more likely to be adopted unsuccessfully (i.e., they were more likely to be relinquished) (OR^1^, 1.8; 95% CI^2^: 1.3–2.5).

The other four characteristics for which there was evidence was found in the literature pertaining to relinquishment. “Financial means” (9, 20), “living in a flat or apartment” (9, 15), “no adopters <21 years old” (13, 26), and “no kennels or outside buildings/dog must live indoors” (24) were all statistically associated with an increased risk of relinquishment. An additional six studies mentioned one or more of these characteristics (4, 10, 16, 24, 28, 31), but they did not provide statistical evidence of an increased risk for relinquishment associated with them.

Patronek et al. (9) reported that compared with households that had an annual income of >75,000 USD, dogs in those with annual incomes of <40,000 USD were associated with a significantly increased risk for relinquishment, and households with incomes <20,000 USD were associated with the greatest risk of relinquishing a dog (OR, 4.43; 95% CI: 2.23–8.81). It should be noted when considering the annual income amounts that Patronek et al. (9) was published over 20 years ago, so the figures may not be representative of today. Dolan et al. (20) reported that dog owners who were on public assistance were statistically more likely to relinquish a dog (OR, 2.3; CI: 1.1–4.9). The study also reported that 71% (115/162) of surrendering owners stated that cost (i.e., inability to pay for some dog care) was either a primary or secondary factor in their decision to relinquish their dog. Patronek et al. (9) reported that 5.6% (16/285) of surrendering owners lived in an apartment and the study concluded that living in an apartment is associated with an increased risk for relinquishment (OR, 2.78; 95% CI: 1.36–5.63). Mondelli et al. (15) noted a relationship between accommodation type and adoption length; adopters living in apartments kept their dog for a statistically significantly shorter period of time than those living in a house^3^. Diesel et al. (13) reported that dogs adopted by people <25 years old were statistically more likely to be rehomed unsuccessfully (OR, 2.9; 95% CI: 1.7–5.0) compared to those adopted by people >50 years old. New et al. (26) noted that surrendering owners were significantly more likely to be <50 years old, and they were most likely to be 20–24 years old (OR, 10.3; 95% CI: 6.9–15.8), followed by <20 years old (OR, 7.7; 95% CI: 4.6–13.0). Kwan and Bain (24) reported that relinquishing owners were statistically more likely to keep their dogs outside 100% of the time compared with continuing owners (i.e., a control sample of owners who are not relinquishing their dog; *p* = 0.03).

An additional seven “most important” characteristics were mentioned in the literature, but such studies did not provide statistical evidence of an increased relinquishment risk (4, 10, 11, 13, 15, 16, 18, 21, 28, 31). One of these was *awareness of needs*, which was itself a theme and a “most important” characteristic; it was mentioned in the literature in terms of an awareness of the amount of time required for a dog's care, which could be a component of *awareness of needs* (4, 11, 13, 15, 28, 31). Another theme, *experience*, which was also a theme itself, but not a “most important” characteristic, was mentioned in the literature pertaining to a dog's quality of life. Marinelli et al. (25) reported that the level of attachment, which the study used as an indicator of a dog's quality of life, was statistically stronger between dog and owner if the owner had previous experience with pets. The study did not specify the quality of care or experience that the owners had in their sample, so it is not known whether such owners had what would be qualified as a “good pet history” (a “most important” characteristic), and thus this cannot necessarily be considered as evidence for the inclusion of this characteristic. The two “most important” characteristics that comprise the *education* theme did not appear to be mentioned in the literature. Characteristics relating to *dog information* and *dog reaction* were excluded from this portion of the analysis, as they did not contain any of the “most important” characteristics.

### RQ4: How Are Adopter Screening Assessments Implemented at a Practical Level?

Seventy-five respondents (91.46%) provided information on how responses gathered from the adopter screening process are scored. Forty respondents (48.78%) used a pass/fail scoring system. Including these respondents, 49 (65.33%) appeared to have specific criteria that they either require or highly value. The way in which the remaining respondents scored or judged adopters' responses can be divided into two categories:

- those who use the information to match the adopter to a specific dog, and
- those who equally value or collectively assess all of the information they gather from an adopter to gain an overall picture.

Of the 75 respondents who provided information, 15 (20%) scored responses in the former manner; they are focused on a specific dog's needs and if the potential adopter appears to be able to meet these needs. Depending on a given dog's needs, they may more highly value some criteria over others. Of the 75 respondents who provided information, 11 (14.67%) claimed to equally value or collectively assess all of the information they gather to gain an overall picture. They may also be using this information to help them match a dog to the adopter.

Of the 81 respondents that always or sometimes used home visits as an adopter screening method, 25 (30.86%) provided information on who conducts their home visits. Ten respondents (40%) reported that their home visits are conducted only by volunteers, and seven respondents (28%) reported that they are conducted only by trained home checkers, but did not clarify whether such individuals are staff members or volunteers. One respondent who used volunteers specified that they are trained volunteers, and another respondent specified that they are experienced volunteers. The responses for the remaining eight respondents (32%) were grouped into an “*other*” category, as they could not be definitively included in the preceding two categories. The *other* category included, though was not limited to, a representative from the respondent or another ADCH member, a member of management, and a trustee.

Of the 69 respondents that always or sometimes used interviews, 20 (28.99%) provided information on who conducts their interviews. Of those, 13 respondents (65%) reported that only staff conduct their interviews, five respondents (25%) reported that only volunteers conduct them, and two (10%) reported that they are conducted either by staff or volunteers.

The greatest level of standardisation in the three adopter screening methods was in self-administered questionnaires; they were completely standardised for 64 respondents (95.52%). The least level of standardisation was in interviews; they were completely standardised for only 13 respondents (18.84%), but were often partially standardised (29 respondents, 42.03%) (See Table 7). For the respondents that conducted completely standardised home visits, 15 (46.88%) required the individual who is conducting the home visit to make subjective judgements of the suitability of the adopter and their environment (e.g., a visit form that includes the item, “Your assessment of their suitability to adopt [this breed].”).

**Table 7:** Standardisation of self-administered questionnaires, pre-adoption home visits, and interviews.

	**Standardised**	**Somewhat standardised**	**Unstandardised**	**No information**
Self-administered questionnaires	64/67 (95.5%)	0	3/67 (4.5%)	0
Pre-adoption home visits	32/81 (39.5%)	17/81 (21.0%)	8/81 (9.9%)	24/81 (29.6%)
Interviews	13/69 (18.8%)	29/69 (42.0%)^a^	15/69 (21.7%)	12/69 (17.4%)

a *For organisations that use a form as part of the interview, this refers to any alterations of any magnitude to it*.

## Discussion

A total of 37 “most important” characteristics were identified, and 31 could be used to prevent a potential adopter from adopting a dog. However, the academic literature does not provide an abundance of evidence to support this. In fact, evidence could only be found in the literature for eight of the characteristics (see Table 6). Four of those characteristics were associated with both an increased risk for relinquishment and a risk to human safety in the relevant literature, and they all were part of the *family* theme pertaining to ages of children in the home. The other four characteristics, for which there was evidence to justify their inclusion in adopter screening assessments, were associated only with an increased risk for relinquishment. This type of evidence comes indirectly in the form of prevalence studies associated with relinquishment (see Table 6; note that depending on the policies of the organisations that participated in these studies or on the design of the study, surrendering owners could have provided multiple reasons for relinquishment; the wider issue of data quality associated with owner report is discussed further below), but mainly from studies that examine risk factors for relinquishment. The latter are often conducted retrospectively based on either the reasons for relinquishment provided by surrendering owners, or the descriptive characteristics of the surrendering owners. Being correlational studies, their direct causal importance cannot be established. Additionally, while statistical evidence was found in the literature for eight of the characteristics, there is variation in terms of the magnitude of effect for this evidence, such as in the odds ratios reported, and this should be noted. There is also variation in terms of the nature of the studies themselves in which this evidence was reported, such as in their sample sizes and study design (see Table 1). Future research could investigate the importance of these variables to the usefulness or gravity of the studies' results.

No evidence could be found in the literature to support the inclusion of any of the characteristics on the basis of a dog's quality of life or overall wellbeing. However, this does not necessarily mean that these characteristics are irrelevant to a dog's quality of life; it just may be that no scientific research has investigated such potential relationships yet. Undoubtedly from an ethical perspective, rehoming organisations need to consider more than just whether or not a dog is likely to be relinquished. When considering the placement of a dog in a new home, a dog's quality of life and overall wellbeing, which might include factors such how long per day a dog is left alone or even whether there are young children in the home, may also be important. How to assess quality of life in dogs is even more scientifically challenging, and thus perhaps not surprisingly has largely not been considered specifically in this context in the literature to date. This would be a useful focus for future academic research.

Nearly half of the “most important” characteristics (45.95%) were found to be around the *accommodation* theme, which indicates considerable attention is paid to a potential adopter's physical environment, especially a garden and the type of building and its features. Considering how highly such factors seem to be valued and how many of them would lead an adopter to be deemed unsuitable to adopt a dog, it might seem reasonable to suppose that the literature should support this (i.e., these are established risk factors for relinquishment, are associated with an increased risk to human safety, or are associated with a dog's quality of life). However, this is not generally the case. Although four characteristics considered “most important” in this theme are mentioned in the literature [“living in a flat or apartment,” “landlord issues,” “the presence of a garden,” and having a “secure garden” (4, 9, 10, 15, 28)], only one, “living in a flat or apartment”, is reported to be statistically associated with an increased risk for relinquishment (9, 15). Moreover, none of the characteristics in this theme are statistically associated with a risk to human safety or a dog's quality of life in the literature, nor were they even mentioned in the relevant literature. Mondelli et al. (15) simply noted a relationship between accommodation type and adoption length; with adopters living in apartments keeping their dog for a statistically significant shorter period of time than those living in a house; whereas Patronek et al. (9) reported that living in an apartment was associated with an increased risk for relinquishment (OR, 2.78; 95% CI: 1.36–5.63). However, the same study also reported that living in a mobile home is an even greater risk factor for relinquishment (OR, 3.54; 95% CI: 1.87–7.10). Perhaps curiously, this latter factor was not considered by any respondents as a reason to bar a potential adopter from adopting a dog. Other studies simply report the prevalence of certain related risk factors but do not evaluate their statistical importance. Thus, Salman et al. (28) found that “inadequate facilities” was a reason provided for 4% of dogs relinquished, which was the seventh most common reason reported, but do not elaborate further on what inadequate facilities refers to; by contrast, Marston et al. (16) found that of the 31.9% (996/3,123) of dogs relinquished for owner-related reasons, 40.4% (403/997) were relinquished due to accommodation and moving. This was the most common owner-related reason, and that was the most common classification of reasons given, but the proportion of surrendered dogs for which reasons were not reported was even greater (34.26%) (1,070/3,123).

Several of the factors referred to by respondents in the current study are quite specific (e.g., “no laminate flooring in main living areas of house”), and do not appear to have been considered at all in the scientific literature or necessarily have any scientific or logical basis. It seems that many of the factors considered by rehoming organisations are based purely on some form of personal opinion, with little or no consideration given to potential mitigating factors (such as the use of mats to improve grip in the case of laminate flooring).

The scientific evidence to support the importance of many factors, like the presence of a garden, may also be challenged when the proportion of dogs kept in association with and without the risk factor is unknown. Taking the presence of a garden as an example, there are two points to consider in relation to the evidence for the importance of this factor. First, there is a deficit of research that has specifically investigated the relationship between the presence of a garden and whether or not a dog is relinquished, although Mondelli et al. (15) concluded that having a house with outdoor space positively influenced the length of adoption, and Diesel et al. (4) noted that the majority of dogs being surrendered had a garden or yard (91.2% [2,560/2,806]), and only 5.9% (166/2,806) did not have one. Similarly, no research has investigated the relationship between the presence of a “secure garden” and whether or not a dog is relinquished. By contrast, as mentioned previously, there is evidence that living in an apartment is a risk factor for relinquishment (9, 15), but this is a very crude proxy measure for the presence of a garden. Characteristics relating to a garden may be included in assessments as they are believed by rehoming organisations to affect a dog's quality of life, but once again, there is a lack of research that has specifically investigated whether there is a relationship between a dog's quality of life and the presence of a garden in their home. Indeed, it might be that those without a garden walk their dogs more and so provide a more enriched life for their dog. Second, in those studies that have identified broad categories of reasons for relinquishment (that may include having or not having a garden), the proportion of dogs relinquished for reasons related to a garden does not appear to be very high, so it may not be a very important factor to consider in any case.

Within the *family* theme, there is evidence (13, 19, 29, 30, 32) to support the inclusion of characteristics relating to the age of children in the household, both in the literature pertaining to relinquishment risk and human safety risk. More support this than any of the other “most important” characteristics. “No children <5 years old” and “no young children” were recurring issues of concern. However, most of these studies relating to relinquishment risk report descriptively. The exception to this Diesel et al. (13) who found that households with children <13 years old were at an increased risk for relinquishing a dog (OR, 1.8; 95% CI: 1.3–2.5). Similarly, while there is a greater body of evidence to support the inclusion of these characteristics on the basis of human injury risk, studies reported that children *over* the age of 5 years old were still at an increased risk for injury compared to other age groups. Shuler et al. (30) reported that the rate of dog bites sustained by boys aged 5–9 years old was significantly higher than the rate of other male age categories (*p* = 0.01), and had the highest incidence rate of any other sex/age category (178 per 100,000 children). In an analysis of people seeking treatment for dog bites in hospital emergency departments, Weiss et al. (32) reported that the incidence of dog bites sustained by children 0–9 years old was significantly higher than for any other age group of children or adults in the study, and especially boys aged 5–9 years old had the highest rate, 60.7 emergency department visits per 10,000 people (95% CI: 34.8–86.6). The relationship with age is further complicated by the level of supervision that should be part of responsible dog ownership, and it might be that this is more important than age *per se*, i.e., the apparent risk of age is actually dependent on the need for supervision, but this does not appear to have been investigated systematically. Indeed, the presence of children of any age in the home might impact on a dog's quality of life. Marinelli et al. (25) reported that the absence of children of any age in the home significantly *increases* owner attachment to the dog, which the authors claim is an indicator of good quality of life for the dog. Thus, the scientific evidence would suggest that it might be more rational for many organisations to not only expand the age group of children they enquire about in adopter screening assessments, but also investigate the supervision available and attitudes to this. This would mean that they should not prohibit households with children of any given age from adopting a dog, but rather they should recognise the potential increased risk, and see if this can be mitigated.

All five of the characteristics that comprise the theme relating to *work/lifestyle* were reported by some respondents to be factors that could lead to a potential adopter being unable to adopt a dog, yet there is no direct scientific evidence to support this. Four of these characteristics (“amount of time dog is alone during day,” “must not be alone for >4 hours,” “must not be alone for >5 hours,” and “lifestyle changes”) are mentioned in the scientific literature (4, 11, 13, 16, 28, 31), but only at a descriptive level, with none of the studies calculating an increased risk for relinquishment associated with these characteristics. For example, Diesel et al. (4) simply reported that nearly a quarter of relinquished dogs were left alone for 4–6 consecutive hours. Whilst some of these characteristics might at least theoretically be associated with an increased risk for relinquishment, no studies appear to have specifically evaluated this yet. Rehoming organisations seem to place a lot of emphasis on this theme, and there is growing concern over the welfare of “home alone” dogs, so this area should perhaps be a priority for future research.

In relation to *expectations*, of all the characteristics identified, only “financial means” appears to be referenced in the literature. Its importance is highlighted descriptively in four studies (4, 16, 28, 31), but Patronek et al. (9) and Dolan et al. (20) statistically quantified the relationship between relinquishment and annual household income ranges. Patronek et al. (9) reported that compared with households that had an annual income of >75,000 USD, dogs in those with annual incomes of <40,000 USD were associated with a significantly increased risk for relinquishment, and households with incomes <20,000 USD were associated with the greatest risk of relinquishing a dog (OR, 4.43; 95% CI: 2.23–8.81). It should be noted that this study was published over 20 years ago, and so the actual annual income figures may not be representative of today's salaries, but it does seem to reflect a trend that deserves consideration and replication. Dolan et al. (20) reported that dog owners who were on public assistance were statistically more likely to relinquish a dog (OR, 2.3; CI: 1.1–4.9). We suggest there are at least two issues that need to be considered with respect to the relationship between income and relinquishment risk and how this information might be used to determine whether a potential owner has the financial means to care for a dog (the reason widely volunteered for making this enquiry by the organisations surveyed). First, if income ranges are used [as in (9)], then it must be recognised that these are dependent on things such as local cost of living and so it is difficult to generalise. Second, “financial means” is a complex concept dependent on a myriad of factors, many of which depend on the priorities of the individual; it is therefore challenging to objectively quantify. The relationship between income levels and allocation of income for the care of the dog is not a direct one and further investigation into other correlates of socio-economic status may be of value in helping to mitigate against the associated risk. Thus, it makes little sense for “financial means” to be a critical characteristic or objectively used threshold within adopter screening assessments. Indeed, we suggest that there are other *expectations* that have been noted in the literature, which may be more important to assess. Patronek et al. (9) reported that if the work caring for a dog was more than expected, this statistically significantly increased the risk for relinquishment (OR, 5.77; 95% CI: 3.25–10.25). Moreover, the study noted that a greater proportion of owners who had obtained their dog from a shelter reported that their dog had been more work than expected compared to owners who obtained their dog from other means. The importance of this factor is further supported by Diesel et al. (13), who also reported that owners who found the work and effort in caring for a dog to be more than expected had a statistically significantly increased risk for relinquishing their dog (OR, 9.9; 95% CI: 4.1–24.6). The importance of this is reinforced by the finding by Diesel et al. (4) that 35.9% (1,009/2,806) of surrendering owners reported that their dogs were more work than expected. Likewise Scarlett et al. (11) found that “poor preparations and inappropriate expectations” was one of the most common classes of reasons for relinquishment cited and accounted for 13.5% (276/2,045) of dogs. A deficit in owner knowledge about dog care and behaviour, alongside impulsive choices, may contribute to unrealistic owner expectations concerning the time required to look after a dog so that problems do not develop (11, 26). Thus, we suggest that perhaps it would be useful if adopter screenings paid greater attention to potential adopter knowledge of dog behaviour and care, as well as the time and resources involved in the care of a dog.

In relation to the *demographics* theme, only one study (28) considers the specific age range, mentioned in our study (“no adopters <21 years old”), but the study did not statistically assess whether adopters in that age range were more likely to relinquish a dog, so its importance remains unknown. However, Diesel et al. (13) did report that dogs adopted by people <25 years old were statistically more likely to be rehomed unsuccessfully (OR, 2.9; 95% CI: 1.7–5.0) compared to those adopted by people >50 years old. Similarly, New et al. (26) noted that surrendering owners were significantly more likely to be <50 years old, and they were most likely to be 20–24 years old (OR, 10.3; 95% CI: 6.9–15.8) followed by <20 years old (OR, 7.7; 95% CI: 4.6–13.0). Indeed, both Shore (10) and New et al. (26) noted that the majority of surrendering owners were in their mid 20s to late 30s. We suggest that rather than age, *per se*, be used to screen owners, that future attention and research should be focused on potential correlates of age and the risk of relinquishment, such as the provision of sufficient resources for a healthy owner-dog relationship and the presence of support systems to assist with the care of the dog as necessary.

The current lack of scientific evidence to support the importance of characteristics relating to the *education* or *experience* of an owner is perhaps surprising. None of the characteristics considered “most important” by some organisations, i.e., “knowledge of breed needs,” “knowledge of force free training,” and “other pets must be neutered and vaccinated/good pet history,” appear to have been considered in the published research to date. Given our earlier comments about the importance of owner expectations, we consider research into an appropriate knowledge base of potential adopters (however obtained) to be a high priority in this field. This is closely related to the final theme identified, “*awareness of needs*,” which was identified by one respondent as a “most important” characteristic in itself, that could lead to an adopter being deemed unable to adopt a dog. The respondent did not elaborate further, and we suggest that this is a critical element of responsible ownership, regardless of its effect on relinquishment. It might include factors such as an awareness of the amount of time required for the dog's care, which has been mentioned in six studies (4, 11, 13, 15, 28, 31) although its significance has not been quantified. Aside from the evidence (or lack thereof) found in the academic literature to justify including the “most important” characteristics as part of the adopter screening assessments, it is possible that other justifications may exist.

One other consideration that should be made when evaluating the nature of the “most important” characteristics is the role of social justice within the adopter screening process. For example, some of these characteristics may be associated with particular races or socioeconomic classes of potential adopters (e.g., the presence of a garden, the amount of time dog is alone during day, financial means). Therefore, a bias related to such social justice issues may intrinsically exist within some organisations' adopter screening processes based on the characteristics that adopters are preferred to or required to possess in order to adopt a dog. Additional research is needed to fully investigate the role of social justice within organisations' adopter screening policies and procedures.

It is worth highlighting how heavily owner reporting is relied on in studies that report or investigate reasons for relinquishment, and that there are a myriad of factors that can affect the quality of this information. This ranges from inaccurate memory, the desire to provide socially acceptable responses [e.g., (34, 35)], and error associated with the emotionally-charged experience of surrendering a dog. A few studies (36, 37) have evaluated the quality of owner reporting in this context, and these indicate that this may be a problem, although the findings are inconsistent. Segurson et al. (37) found that relinquishing owners who believed that their questionnaire responses were confidential reported significantly more often that their dogs displayed owner-directed aggression and fear of strangers than those who believed their responses were not confidential. In contrast, Duffy et al. (36) reports that relinquishing owners did not give unreliable or biased responses on a behavioural evaluation regardless of the confidentiality of their responses, although the reliability of such behavioural evaluations might be questioned.

It should be noted that whilst the adopter screening methods evaluated in this study are widely used by many rehoming organisations, there is a growing trend amongst organisations to move away from stringent adopter screening policies and procedures, and instead employ a conversation-based approach. The Humane Society of the United States advocates for this type of approach in which formal procedures, such as adoption applications, are viewed as barriers that could prohibit a potentially successful adoption. Instead, a dialogue or conversation between the potential adopter and a representative from the organisation is created to help find the best match between dog and adopter (38). It is possible that even though respondents in this study did not use the specific terminology, some are actually using a version of a conversation-based approach, namely those that do not use a pass/fail scoring system or more highly value specific adopter criteria. This may also have been the case for respondents who reported having somewhat standardised or unstandardised screening methods; in fact, it may explain to some extent why interviews were the least standardised of the three screening methods. This conversation-based approach does not yet seem to have received an abundance of attention in the scientific literature, but Weiss et al. (39) did investigate whether this method compared to a more traditional, policy-based approach affected the quality of care and attachment between adopters and their pets. The study's authors concluded that the quality of care and level of attachment did not substantially differ between the two approaches. Because there is a recent shift in some rehoming organisations to a conversation-based approach to match dogs to adopters, this is an area that is deserving of additional scientific research.

Our work also highlights concerns over the quality of the practical execution of adopter screening assessments, associated with both subjectivity and lack of standardisation. Based on the low levels of standardisation in home visits and interviews coupled with the range of people who conduct both, it is likely that there are frequent and possibly grave inconsistencies in their practical execution. Moreover, of the organisations that have completely standardised home visits, a considerable proportion (46.9%) requires whoever is conducting the home visit to make subjective judgements of the suitability of the adopter and their environment. Because one person may conduct the home visit and another person may conduct the rest of the screening process, there is further opportunity for inconsistencies in the overall assessment. This is further cause for concern in terms of the quality of information being used to answer the question “Can this dog be rehomed to you?”

## Conclusion

Our findings indicate that organisations invest considerable resources into screening potential adopters, but there seems to be little scientific, and in some cases logical, rationale for some of the factors investigated. The scientific evidence that is available supports only eight of the 37 characteristics identified as “most important,” and even this might be considered relatively weak, since it comes largely from studies that have evaluated owner-reported reasons for relinquishing their dog or associations, rather than causal relationships, with the characteristics of these owners or associations between medical records (as a proxy of human injury risk, despite obvious biases in who is likely to seek help). Although it must be acknowledged that the purpose of including at least some of the screening items is to ensure a good quality of life or overall wellbeing for a dog, here too there is a considerable lack of relevant research, and even so the rationale for simple generalisations is highly questionable even on the basis of a “precautionary principle.” There are further concerns relating to the quality of the assessment processes, which show little evidence of reasonable quality control measures. We question the validity of including many of the factors assessed in a pass/fail manner, which could lead an adopter to be deemed unsuitable to adopt a dog, since even those with evidence to support them may be indirectly causally linked. Until the necessary research is conducted, it could be argued that from a pragmatic perspective, organisations should consider relaxing their screening processes and associated criteria, and instead focus their resources on ensuring owners are fully prepared for the changes in their life associated with the inclusion of a new dog in their home and supporting them as necessary post-adoption.

## Data Availability Statement

The raw data supporting the conclusions of this article will be made available by the authors, without undue reservation.

## Author Contributions

KG and DM identified the need for the study based on the lack of relevant academic research. All authors then conceived of the nature and design of the study. KG recruited participants for the sample and collected data. KG and DM qualitatively analyzed the data. KG wrote the first draft of the manuscript. DM contributed to the writing of multiple sections of the manuscript. All authors read and edited multiple drafts of the manuscript, and approved the final version.

## Conflict of Interest

The authors declare that the research was conducted in the absence of any commercial or financial relationships that could be construed as a potential conflict of interest.

## Footnotes

^1^The abbreviation *OR* refers to odds ratio.

^2^The abbreviation *CI* refers to confidence interval.

^3^This study did not report its statistical values.
